# Management precautions for risk of obesity are necessary among infants with PKU carrying the rs113883650 variant of the LAT1 gene: A cross-sectional study

**DOI:** 10.1371/journal.pone.0264084

**Published:** 2022-02-17

**Authors:** Miroslaw Bik-Multanowski, Bozena Didycz, Kinga Bik-Multanowska

**Affiliations:** Department of Medical Genetics, Jagiellonian University Medical College, Krakow, Poland; Sohag University Faculty of Medicine, EGYPT

## Abstract

Patients with phenylketonuria (PKU), an inborn error of phenylalanine metabolism, require consistent treatment to avoid the brain toxicity caused by hyperphenylalaninemia. The treatment consists of life-long use of a low-phenylalanine diet, which aims at decreasing hyperphenylalaninemia and maintaining blood phenylalanine concentration in a safe range. Problems with balancing diet can result in suboptimal treatment outcomes; however, recent findings suggest that genetic alteration of the transport of phenylalanine might result in an additional health burden. We assessed the effect of a common variant (rs113883650) of the *LAT1(SLC7A5)* gene, which encodes the main transmembrane phenylalanine transporter, on the development of overweight in 54 infants with PKU who received standard therapy and adhered well to therapeutic prescriptions, and in 55 infants with a milder disease form—the so-called mild hyperphenylalaninemia (MHP), which does not require treatment. We found that infants with PKU—carriers of the rs113883650 variant had significantly higher Body Mass Index (BMI) at 1 year compared to PKU infants without the variant (mean BMI Z-Score of +1.15 SD vs -0.15 SD, respectively; t(52) = 5.25, p = 0.00005). Conversely, no significant BMI differences were detected in the subgroups of infants with MHP (t(53) = 1.15, p = 0.25). Additionally, high BMI in infants with PKU—carriers of the rs113883650 variant positively correlated with high variability of their blood phenylalanine levels (r(52) = 0.42, p = 0.002). It should be noted that this is an observational study, which does not determine causation. Nevertheless, our findings show that the rs113883650 variant of the *LAT1* gene may be a risk factor for overweight in properly treated infants with PKU. Management precautions should be taken to prevent the development of overweight and obesity.

## Introduction

Phenylketonuria (PKU; OMIM #261600) is a classic example of a treatable genetic condition. The disease results from an inherited dysfunction of the enzyme phenylalanine hydroxylase, leading to hyperphenylalaninemia with blood phenylalanine concentration above 0.12 mmol/l. For patients with PKU, the treatment goal is to reduce severe hyperphenylalaninemia, which is toxic to the brain. The therapeutic, life-long low-phenylalanine diet should be initiated in neonates with hyperphenylalaninemia, whose blood phenylalanine concentration exceeds 0.36 mmol/l [1, 2], whereas those with mild hyperphenylalaninemia (MHP), in whom blood phenylalanine does not exceed 0.36 mmol/l, do not require any treatment.

The adequateness of the low-phenylalanine diet in patients with PKU requires measurements of blood phenylalanine concentration with subsequent adjustment of the diet if phenylalanine level falls outside the recommended range of 0.12–0.36 mmol/l [2]. Blood phenylalanine concentration should be frequently measured during the first year of life since the daily tolerance of phenylalanine decreases during this period, reflecting the decreasing velocity of body growth. However, the interindividual differences in body growth dynamics make it virtually not possible to predict all phenylalanine fluctuations, especially that the feeding of the patient cannot be fully controlled by the medical team at the patient’s home, where commonly occurring factors such as mild infections, dyspepsia episodes or teething problems may result in increased catabolism with subsequent “spikes” of blood phenylalanine [2]. The phenylalanine level should be normalised in such cases as quickly as possible, but in patients with a complete metabolic block (“classic” PKU), it often takes several days until the phenylalanine concentration returns to the safe range of 0.12–0.36 mmol/l. Based on our clinical experience and the observations of other authors, the length of periods of insufficient control of hyperphenylalaninemia usually does not exceed 20% of the time during the first year of life of a patient [3, 4]. Nevertheless, parental problems with adherence to the therapeutic recommendations could additionally increase the length of such periods.

Uncontrolled hyperphenylalaninemia can result in brain dysfunction and the development of secondary health problems, such as being overweight [5]. However, additional genetic factors can also be important for treatment outcomes. We recently demonstrated that carriership of a common variant rs113883650 of the *LAT1(SLC7A5)* gene, encoding the major transmembrane transporter of phenylalanine, may affect the concentration of this amino acid in the brain in teenagers and young adults with severe hyperphenylalaninemia [6]. We also demonstrated the presence of this variant of the *LAT1(SLC7A5)* gene, which occurs in approx. 45% of the population in Europe correlates with becoming overweight in infants with severe PKU [7]. However, although all the patients from our previous study were frequently monitored, episodes of increased blood phenylalanine occurred in almost all of them and lasted often longer than 20% of the first year of life, suggesting potential parental problems with treatment adherence. Such problems could contribute to the development of overweight. Therefore, in the present study, we aimed to answer two research questions:

- Is the rs113883650 variant a risk factor for overweight in well-controlled infants with PKU, in whom blood phenylalanine concentration remains below 0.36 mmol/l for most (at least 80%) of the first year of life?
- Are patients with mild hyperphenylalaninemia (MHP), in whom blood phenylalanine concentration is constantly elevated but remains in the recommended “safe” range 0.12–0.36 mmol/l without dietary treatment, also at risk of overweight?

An affirmative answer to any of the above questions should result in revising the current standards of medical care for patients in PKU to better prevent overweight development in a large group of youngest patients.

## Patients and methods

In this cross-sectional, retrospective study, we aimed to assess the influence of the rs113883650 variant of the *LAT1* gene on weight gain in two cohorts of infants with inherited hyperphenylalaninemia: patients with PKU who receive dietary treatment and effectively maintain the recommended blood phenylalanine concentrations and infants with MHP, in whom constantly elevated blood phenylalanine can be observed, but it remains < 0.36 mmol/l and no diet is recommended.

The study was conducted at the Department of Medical Genetics, Jagiellonian University Medical College, Krakow, Poland.

For the purpose of estimation of the minimal sample size in the present study, we analysed the previously observed [7] effect of the carriership of the rs113883650 of the *LAT1* gene variant on Body Mass Index (BMI) in infants with PKU during their first year of life. We considered that the average BMI at the age of one year was approx. 6% higher in the group of carriers of the targeted variant than in the group of wild-type individuals. We also considered the ratio of carriers of the rs113883650 variant and of wild type individuals, which equals approx. 0.8 in our population [6, 7]. The subsequent estimations of sample size were performed using an online calculator (www.clincalc.com). It revealed that statistically significant results could be obtained if at least 52 patients (carriers and wild-type individuals) were recruited for the study (1-alpha = 95%; 1-beta = 80%) in each cohort (patients with PKU and those with MHP).

To recruit patients for the study, we analysed the medical records of patients with MHP and PKU born in 2001–2020 and who were followed up in our centre. Based on this analysis, we first selected a group of 55 consecutive patients with MHP, in whom blood phenylalanine remained in the range 0.12–0.36 mmol/l without using a diet. Next, we selected a cohort of 54 consecutive patients with PKU without apparent treatment adherence problems in infancy, in whom the blood phenylalanine concentration was within the recommended limit of 0.12–0.36 mmol/l for at least 80% of the first year of life. We excluded patients with low birth weight (<2500 g) and those suffering in infancy from any additional disease that could affect the physiologic weight gain process (e.g., chronic diarrhoea or gastrointestinal reflux).

All the selected patients with PKU were treated using a standard dietary protocol: recommended blood phenylalanine concentration of 0.12–0.36 mmol/l, a daily protein intake of approx. 2.1–2.4 g/kg of body weight. The patients were admitted to the hospital immediately after receiving a positive result of the neonatal screening test in the second week of life. Dietary treatment was introduced immediately after confirming hyperphenylalaninemia in the hospital laboratory, and blood phenylalanine levels were lowered to the recommended range during the third week. The process of diet introduction was completed in the third or at the beginning of the fourth week of life in all assessed patients. Subsequently, the patients were discharged from the hospital.

Individually calculated, necessary amounts of breast milk or standard infant formulas combined with phenylalanine-free formulas were used to feed infants with PKU. Before feeding in breastfed infants, the individually calculated, appropriate amount of breast milk was mixed with phenylalanine-free formula. Breast milk or commercially available standard infant formulas constituted the sources of natural protein in youngest infants, whereas fruits and vegetables (commercially available products for infants with known caloric and protein intake) were added to the diet in the second half of the first year. The metabolic physician and dieticians together consulted every follow-up result of the control blood phenylalanine assessment. However, the daily intake of energy and phenylalanine was monitored by a metabolic dietician, who was also responsible for adjusting the diet on a week-to-week basis, if necessary. The dieticians used standard guidelines for the management of potential dietary issues. Blood phenylalanine concentration was controlled on a weekly/biweekly basis.

For the purposes of this study, we assessed the medical history of all patients and recorded their body weight at birth as well as body weight and body length at the age of one year. Subsequently, we calculated the body mass index (BMI) for every patient at 1 year.

To compare the subgroups of patients regarding their anthropometric data, we utilised the birth weight Z-scores and BMI Z-scores at the age of one year, which are based on the growth charts recommended for the Polish population [8]. We did not calculate BMI at birth since exact body length measurement in newborn babies is not always possible in delivery wards due to the lack of dedicated infant measuring boards. Instead, we focused on birth weight assessment since bodyweight measurement after birth is easy, and neonatologists widely use the result for the assessment of the nutritional status of the baby. On the contrary, we used the BMI Z-Score values at one year since it was shown that it is a good predictor of overweight and obesity in older infants [9]. Moreover, exact measurements of body weight and body length of our patients at the age of one year were conducted in our clinic by the hospital anthropologist (body length was measured using dedicated specialist equipment).

Next, we genotyped all patients for the rs113883650 variant of the *LAT1* gene using direct sequencing. We used the following PCR primers: Forward: CAC CAG CTG CCC TGG GAT GC and Reverse: TGG CCT GAG CCG ACC AAC AG, as described previously [10]. Finally, to answer the research questions, we compared the carriers of the rs113883650 variant and wild-type individuals regarding their BMI Z-scores at 1 year.

For the statistical analysis, we have utilised descriptive statistics, independent samples t-test, Fisher’s exact test and Pearson’s correlation.

The Jagiellonian University’s Ethics Committee approved this study. Written informed consent for participation in the study was obtained from adult patients (>18 years) or parents of younger patients.

## Results

The rs113883650 variant of the *LAT1* gene was detected in 27 out of 54 patients with PKU and in 25 out of 55 patients with MHP. The allelic frequency of the variant in the entire group of study participants equalled 0.28 (we identified 9 homozygous, 43 heterozygous and 57 wild-type individuals).

In patients with PKU, the mean blood phenylalanine concentration in the first year of life after hospital discharge was nearly identical in both subgroups and reached 0.21 (1SD = 0.15 mmol/l) in carriers of the *LAT1* variant (27 cases) and 0.21 (1SD = 0.14) mmol/l in wild-type individuals (27 cases). Treatment adherence was high in all patients on a low-phenylalanine diet, with very few small phenylalanine “spikes” exceeding 0.36 mmol/l. The individual percentage of blood phenylalanine test results within the recommended limit exceeded 80%. It was very similar in the carriers of the rs113883650 variant of the *LAT1* gene and those with a wild-type genotype (mean = 88% in both subgroups) (Table 1).

**Table 1:** Comparison of carriers of the rs113883650 variant of the *LAT1* gene and wild-type individuals in patients with PKU and MHP.

	Carriers of the rs113883650 variant of the *LAT1* gene	Wild-type individuals	Statistical significance
Patients with PKU (on low-phenylalanine diet)			
Number of cases	27	27	-
Gender	14 girls, 13 boys	8 girls, 19 boys	p = 0.16
Birth weight Z-score (SD)	0.04	-0.28	p = 0.25
BMI Z-score at one year (SD)	+1.15	-0.15	p = 0.00005
Mean yearly blood phenylalanine concentration (mmol/l)	0.21 (1 SD = 0.15)	0.21 (1 SD = 0.14)	-
Mean percentage of blood phenylalanine results within the recommended range (%)	88 (1 SD = 5.9)	88 (1 SD = 5.9)	-
Mean caloric intake in the first month of life (kcal/day)	116 (1 SD = 8.7)	120 (1 SD = 5.5)	p = 0.1
Mean caloric intake at the age of one year (kcal/day)	100 (1 SD = 9.1)	108 (1 SD = 6.6)	p = 0.004
Patients with MHP (no dietary treatment)			
Number of cases	25	30	-
Gender	13 girls, 12 boys	14 girls, 16 boys	p = 0.78
Birth weight Z-score (SD)	+0.02	-0.40	p = 0.11
BMI Z-score at one year (SD)	+0.37	+0.14	p = 0.25
Mean yearly blood phenylalanine concentration (mmol/l)	0.20 (1 SD = 0.06)	0.22 (1 SD = 0.06)	-

No significant differences were found between the subgroups of PKU patients-carriers of the rs113883650 variant and wild-type individuals regarding their mean birth weight. The mean birth weight Z-scores reached -0.02 SD and -0.29 SD in carriers and in wild-type individuals, respectively (t(52) = -0.91, p = 0.36). On the contrary, the mean BMI Z-score at one year was much higher in the group of PKU patients carriers of the *LAT1* variant than wild-type individuals. It reached +1.15 SD vs. -0.15 SD, respectively and the difference was highly significant (t(52) = 5.25, p = 0.00005). BMI exceeding the 95th percentile (BMI Z-Score >1.65)—the threshold of obesity in older children [10]–was found in 29.6% (8/27 cases) of carriers and only in 3.7% (1/27 cases) of wild-type individuals.

**Fig 1:**
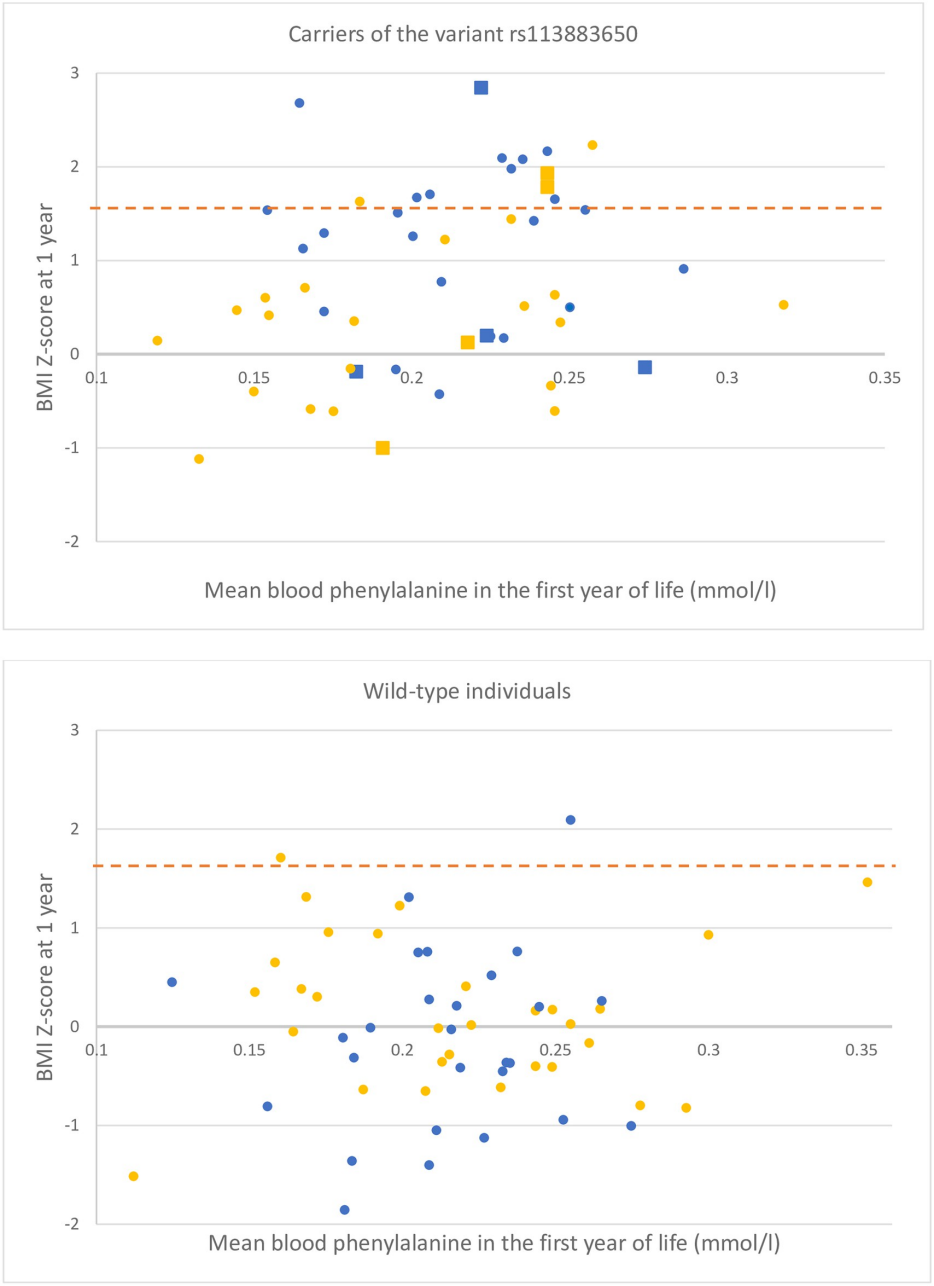
Differences in BMI Z-scores at one year for all infants with (the upper part of the figure) and without the rs113883650 variant of the *LAT1* gene (the lower part of the figure), in the context of mean yearly blood phenylalanine concentrations. In patients with PKU, the mean BMI Z-score at one year reached +1.15 SD and -0.15 SD for the carriers of the *LAT1* variant and wild-type individuals, respectively. The difference between both subgroups was highly significant (t(52) = 5.25, p = 0.00005). For the group of infants without diet, the mean BMI Z-score at one year reached 0.4 SD and 0.14 SD in carriers and wild-type individuals, respectively. The difference was not statistically significant (t(53) = 1.15, p = 0.25). Patients on dietary treatment are marked in blue, whereas those not requiring diet are marked in yellow. Square markers indicate the homozygotes for the *LAT1* variant. The dashed orange line represents the threshold of obesity as defined by the CDC for older children (95th percentile of BMI) [9].

On the contrary, similar calculations in patients with MHP, who did not require dietary treatment, revealed no significant differences. The mean body weight Z-Score at birth reached 0.08 SD and -0.3 SD in carriers and in wild-type individuals, respectively (t(53) = 1.29, p = 0.2), whereas the mean BMI Z-score at one year reached 0.4 SD in carriers and 0.14 SD in wild-type individuals (t(53) = 1.15, p = 0.25) (Table 1, Fig 1).

**Fig 2:**
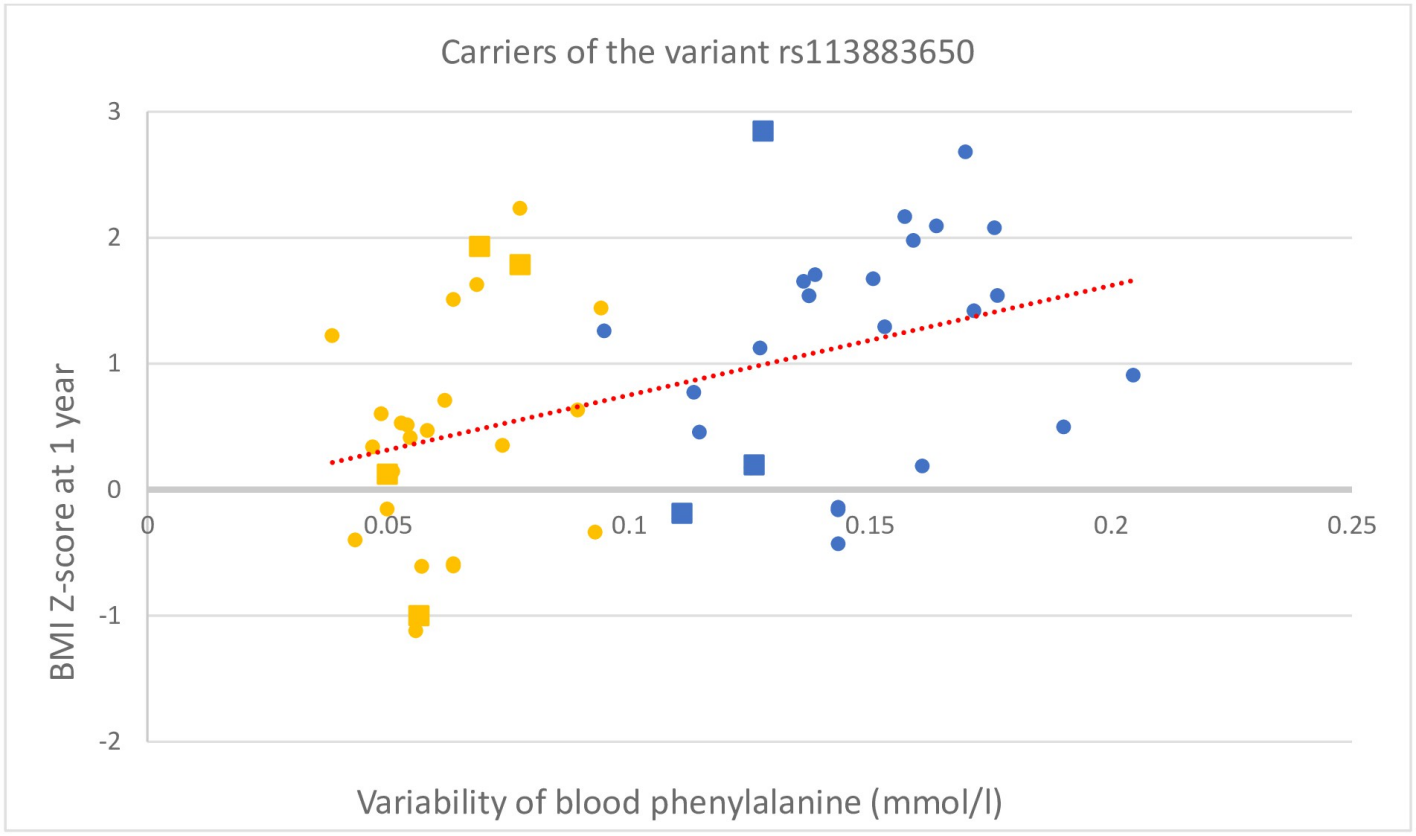
The relation of BMI Z-scores at one year and the variability of blood phenylalanine levels in the first year of life for infants with PKU and MHP—Carriers of the rs113883650 variant of the *LAT1* gene. The variability of blood phenylalanine levels is represented by the SD of all blood test results recorded in the assessed period for a given patient. The BMI Z-scores and blood phenylalanine variability in carriers of the *LAT1* variant were moderately positively correlated, r(42) = 0.46, p = 0.002. Patients on dietary treatment are marked in blue, whereas those not requiring diet are marked in yellow. Square markers indicate the homozygotes for the variant of the *LAT1* gene.

**Fig 3:**
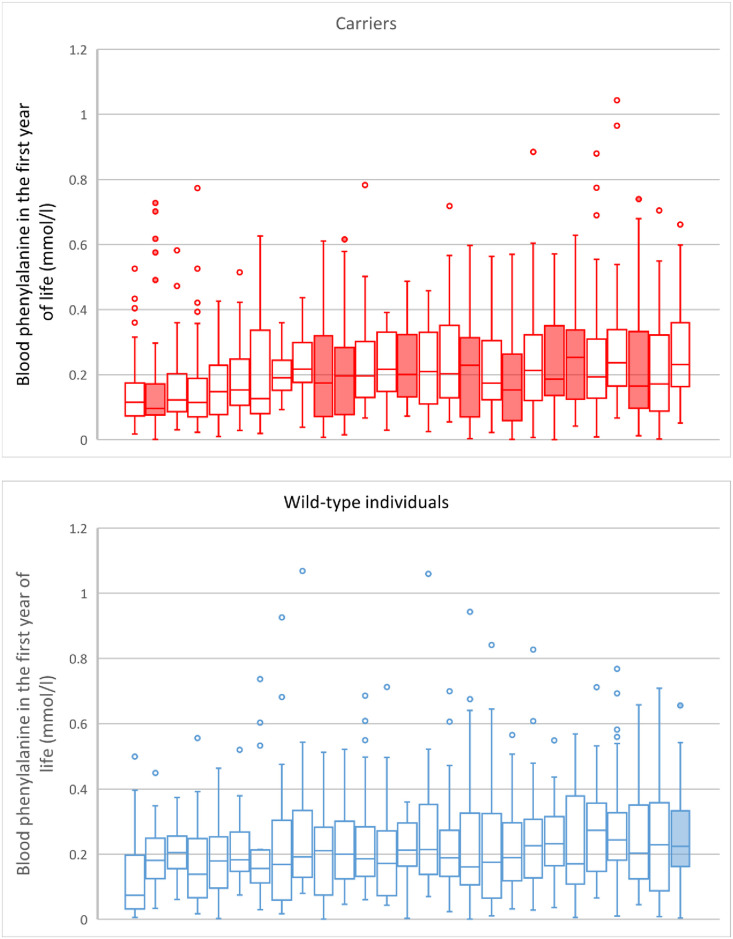
Variability of blood phenylalanine during the first year of life in patients with PKU concerning the carriership of the rs113883650 variant of the *LAT1* gene. The blood phenylalanine values recorded for individual patients are presented in quartiles, separately for the carriers of the *LAT1* variant and wild-type individuals. The mean yearly phenylalanine concentrations in both groups equalled 0.21 mmol/l and the variability, as measured by standard deviation, was also nearly identical (0.14 vs 0.15 mmol/l). Patients with BMI above the 95th percentile (the threshold of obesity in older children according to the Center for Disease Control) are depicted with shaded boxes. Almost all of them carried the rs113883650 variant of the *LAT1* gene.

Further analysis of blood phenylalanine dynamics in the entire subgroup of carriers of the *LAT1* variant with PKU revealed a moderately positive correlation of BMI Z-scores at one year and of blood phenylalanine variability during the assessed period, which was represented by the SD of all recorded blood phenylalanine results in a given patient (r(52) = 0.42, p = 0.002). On the contrary, no significant correlation was found between blood phenylalanine variability and BMI Z-scores in the group of wild-type individuals (r(57) = 0.17, p = 0.2). The mean phenylalanine variability in patients with PKU was nearly identical for carriers of the *LAT1* variant and wild-type individuals (0.15 mmol/l and 0.14 mmol/l, respectively). Additionally, the mean variability was identical for both subgroups of infants with MHP (0.06 mmol/l) (Table 1, Figs 2 and 3).

An additional analysis of the caloric intake in patients with PKU revealed that the mean daily energy intake in the first month was similar in carriers of the rs113883650 variant and wild-type individuals (116 kcal/kg versus 120 kcal/kg, respectively). However, at the end of the first year of life, the difference between both groups increased, and the average caloric intake reached 100 kcal/kg in the carriers and 108 kcal/kg in wild-type patients. The difference was statistically significant (t(52) = -3.09, p = 0.004) (Table 1).

The S1 and S2 Tables show detailed genetic, anthropometric and metabolic data on study participants.

## Discussion

In patients with inborn errors of metabolism, overweight can result from inadequate metabolic control. However, in the present study, we detected a clear tendency to excessive weight gain in infants with a genetically heterogenous but well-controlled PKU, carriers of the rs113883650 variant of the *LAT1* gene. These results align with our previous observations regarding patients homozygous for the most common mutation of the *PAH* gene—p.R408W [7]. Our results suggest that nearly every second infant with PKU may be at risk of becoming overweight or obese if carrying the rs113883650 variant of the *LAT1* gene, even with effective treatment adherence recommended for this age group [3, 4]. However, data from this study suggest that this unwanted effect is absent in untreated infants with mild hyperphenylalaninemia, who are carriers of the rs113883650 variant.

The BMI in infants with PKU—carriers of the rs113883650 variant correlated in our study with high variability of their blood phenylalanine levels (Table 1, Fig 2). Consequently, high BMI and the related risk of becoming overweight represent an unfavourable effect of instability of blood phenylalanine concentration. Our findings are consistent with former observations of negative effects of fluctuations of blood phenylalanine in patients with PKU, such as suboptimal cognitive development and increased level of anxiety [11, 12].

It is not clear whether the variant itself could participate in phenylalanine variability. However, our results do not support such a hypothesis. We observed nearly identical phenylalanine variability in both subgroups of patients with PKU and both subgroups of infants with MHP. It seems that this problem should be addressed in further prospective studies.

The mechanism of becoming overweight in infants with PKU and the effect of carriership of the rs113883650 variant of the *LAT1* gene is unclear. Thus, further basic research (e.g., with the use of cellular models) and confirmatory studies in bigger groups of patients are required. *LAT1* encodes the major transmembrane transporter of large neutral amino acids and thyroid hormones [13]. It can be expected that in the case of severe hyperphenylalaninemia, an excess of phenylalanine competitively inhibits the LAT1-mediated transport of thyroid hormones to the cells. This, in turn, could decrease the basal metabolic rate and increase the risk of becoming overweight in all individuals with long-lasting hyperphenylalaninemia. The carriership of the rs113883650 variant could influence the *LAT1* gene expression due to altering the binding site of the CTCF transcription factor [14], which could potentiate the effect of transport inhibition in thyroid hormones in the long run, even in cases of relatively mild hyperphenylalaninemia.

We observed that at the age of 1 year, the mean daily caloric intake in infants with PKU carriers of the variant of the *LAT1* gene was significantly lower than in wild-type individuals. This is unexpected since the carriers were much heavier. Nevertheless, this finding clearly shows that the excessive weight gain in infants with the rs113883650 variant was not caused by increased energy intake. The reason for the observed difference is not clear, but it might reflect a purposeful dietary intervention of the medical team—a decrease of caloric supply to prevent obesity in infants with excessive weight gain, without prior knowledge on their rs113883650-carriership status.

Being overweight represents a key medical problem in society. Since the mean reported prevalence of PKU is approximately 1:10,000 live births, with a much higher rate in some countries [2] it can be assumed that only in Europe several hundreds of newborns with PKU—carriers of the rs113883650 variant could be at risk of developing overweight every year. If the tendency to increase body weight in infants with PKU persisted, it would be an additional health burden, especially since the metabolic control is rarely perfect in older patients due to individual problems with maintaining the diet.

It should be noted that the functional importance of the rs113883650 variant was not detected in previous genome-wide association studies in the general population [15]. However, this is probably because hyperphenylalaninemia is not observed in persons not affected with PKU. Thus, no overweight due to hyperphenylalaninemia-related alteration of the function of the LAT1 transporter can be expected in healthy persons and the findings of the present study cannot be generalized to healthy populations.

Some limitations of the present study should be mentioned. We did not use any control population and did not assess patients from other metabolic centres and from other countries, which would aid in excluding any unexpected population-specific or region-specific effects. It should also be noted that, this is an observational study, which may indicate associations but not causation. In addition, interindividual differences in feeding behaviour and dietary preferences in patients, as well as social dimension and family dynamics are potential confounding factors. The above issues can be addressed in further confirmatory studies with larger groups of patients. Further research on cellular models is also needed to elucidate the molecular and cellular role of the rs113883650 variant of the *LAT1* gene.

In conclusion, the results of this study are in line with the findings from our previous research and confirm the correlation of the rs113883650 variant of the *LAT1* gene with overweight in infants with PKU. Therefore, in our opinion, routine genotyping of the *LAT1* gene should be considered in all babies diagnosed with PKU, and the therapeutic standards should be reviewed in carriers of the rs113883650 variant, who may need additional medical assistance to prevent overweight.

## Supporting information

S1 Table Mutations of the phenylalanine hydroxylase gene (*PAH*) and anthropometric data of the study participants in the context of their sex and their carriership status regarding the rs113883650 variant of the LAT1 gene. The body weight and BMI Z-scores are calculated based on the growth charts recommended for the Polish population. Homozygotes for the rs113883650 variant are marked with an asterisk.
(DOCX)

S2 Table Blood phenylalanine test results (mmol/l) recorded in participants of the study. (XLSX)
