# Supplementary material for: Management precautions for risk of obesity are necessary among infants with PKU carrying the rs113883650 variant of the LAT1 gene: A cross-sectional study
Source: PLoS One. 2022 Feb 17;17(2):e0264084. doi: 10.1371/journal.pone.0264084 (PMC8853486; doi:10.1371/journal.pone.0264084)
Supplement: S1 Table — The body weight and BMI Z-scores are calculated based on the growth charts recommended for the Polish population. Homozygotes for the rs113883650 variant are marked with an asterisk. (DOCX) [file pone.0264084.s001.docx]

**S1 Table. Mutations of the phenylalanine hydroxylase gene (*PAH*) and anthropometric data of the study participants in the context of their sex and their carriership status regarding the rs113883650 variant of the LAT1 gene.** The body weight and BMI Z-scores are calculated based on the growth charts recommended for the Polish population. Homozygotes for the rs113883650 variant are marked with an asterisk.

| Patient’s number | Mutations of the phenylalanine hydroxylase gene (*PAH*) | Birth weight  (kg) | Birth weight Z-Score (SD) | Body weight at 1 year  (kg) | Body length at 1 year (mm) | BMI  Z-Score at one year (SD) | Phenylalanine:  mean / 1SD  (mmol/l) | Diet | Patient’s number | Mutations of the phenylalanine hydroxylase gene (*PAH*) | Birth weight  (kg) | Birth weight Z-Score (SD) | Body weight at 1 year  (kg) | Body length at 1 year | BMI  Z-Score at one year (SD) | Phenylalanine:  mean / 1SD  (mmol/l) | Diet |
| --- | --- | --- | --- | --- | --- | --- | --- | --- | --- | --- | --- | --- | --- | --- | --- | --- | --- |
| Carriers of the rs113883650 variant of the LAT1 gene variant | | | | | | | | | | | | | | | | | |
| Girls | | | | | | | | | Boys | | | | | | | | |
| 1 | p.R408W/p.R408W | 3.75 | 0.95 | 11.32 | 762 | 1.54 | 0.15 / 0.14 | Yes | 3 | p.R408W/p.R408W | 3.58 | 0 | 12.4 | 808 | 1.12 | 0.17 / 0.13 | Yes |
| 2 | p.R408W/p.R408W | 3.35 | -0.11 | 13 | 777 | 2.68 | 0.17 / 0.16 | Yes | 9 | p.R408W/p.L48S | 3.63 | 0.11 | 12.16 | 796 | 1.26 | 0.2 / 0.09 | Yes |
| 4 | p.R408W/IVS10-11g>a | 3.2 | -0.52 | 11.01 | 760 | 1.29 | 0.17 / 0.15 | Yes | 11 | p.R408W/IVS10-11g>a | 2.7 | -1.96 | 10.26 | 719 | 1.71 | 0.2 / 0.14 | Yes |
| 5 | p.R408W/p.R408W | 3.78 | 1.03 | 9.91 | 751 | 0.46 | 0.17 / 0.11 | Yes | 12 | p.R408W/p.R408W | 3.65 | 0.16 | 9.65 | 760 | -0.43 | 0.2 / 0.14 | Yes |
| 6^*^ | p.R408W/p.Y414C | 3.86 | 1.24 | 9.84 | 774 | -0.19 | 0.18 / 0.11 | Yes | 14* | p.R408W/p.I283F | 3.78 | 0.45 | 12.76 | 770 | 2.84 | 0.22 / 0.13 | Yes |
| 7 | p.R408W/p.P407L | 3.65 | 0.68 | 8.97 | 738 | -0.16 | 0.19 / 0.14 | Yes | 16 | p.R408W/p.R408W | 3.8 | 0.49 | 9.91 | 750 | 0.19 | 0.22 / 0.16 | Yes |
| 8 | p.R408W/p.A403V | 3.45 | 0.15 | 10.56 | 737 | 1.51 | 0.2 / 0.06 | Yes | 17 | p.R408W/p.R408W | 3.09 | -1.09 | 11.15 | 739 | 2.09 | 0.23 / 0.16 | Yes |
| 10 | p.R408W/p.P281L | 3.66 | 0.71 | 12.07 | 782 | 1.67 | 0.2 / 0.15 | Yes | 18 | IVS10-11g>a/IVS10-11g>a | 3.2 | -0.85 | 9.79 | 746 | 0.17 | 0.23 / 0.13 | Yes |
| 13 | IVS10nt-11g>a/p.Y414C | 3.3 | -0.25 | 8.46 | 683 | 0.77 | 0.21 / 0.11 | Yes | 19 | R408W/IVS9nt-2a>c | 3.6 | 0.05 | 12.32 | 780 | 1.98 | 0.23 / 0.16 | Yes |
| 15^*^ | p.R408W/p.R408W | 2.91 | -1.29 | 10.25 | 774 | 0.20 | 0.22 / 0.13 | Yes | 22 | p.R408W/IVS10-11g>a | 4.15 | 1.27 | 11.6 | 766 | 1.65 | 0.24 / 0.14 | Yes |
| 20 | p.R408W/p.R408W | 3.22 | -0.46 | 10.85 | 750 | 1.42 | 0.24 / 0.17 | Yes | 23 | p.R408W/p.R408W | 4.25 | 1.5 | 11.8 | 807 | 0.5 | 0.25 / 0.19 | Yes |
| 21 | p.R408W/p.R408W | 3.74 | 0.92 | 12.9 | 791 | 2.17 | 0.24 / 0.16 | Yes | 26 | p.R252W/IVS10-11g>a | 3.6 | 0.05 | 12.6 | 800 | 1.54 | 0.25 / 0.18 | Yes |
| 24 | p.R408W/p.R158Q | 4.02 | 1.67 | 10.7 | 763 | 0.91 | 0.29 / 0.2 | Yes | 27* | R408W/G307D | 2.5 | -2.4 | 9.51 | 745 | -0.14 | 0.27 / 0.14 | Yes |
| 25 | p.R408W/p.R408W | 2.95 | -1.18 | 12.1 | 768 | 2.08 | 0.23 / 0.18 | Yes |  |  |  |  |  |  |  |  |  |
| 29 | p.L348V/[p.T63P;p.H64N] | 2.84 | -1.48 | 10.11 | 760 | 0.63 | 0.24 / 0.09 | No | 28 | p.A300S/ex5del | 3.4 | -0.40 | 9.39 | 750 | -0.40 | 0.15 / 0.04 | No |
| 30 | p.R408W/p.T380M | 3.71 | 0.84 | 10.3 | 750 | 1.22 | 0.21 / 0.04 | No | 32* | p.R408W/p.V245A | 3.22 | -0.80 | 10.74 | 750 | 1.44 | 0.23 / 0.09 | No |
| 31 | p.I306V/p.D415N | 3.64 | 0.66 | 9.07 | 760 | 0.35 | 0.18 / 0.07 | No | 36 | p.R297H/not identified | 4 | 0.94 | 10.8 | 774 | 0.47 | 0.15 / 0.06 | No |
| 33 | p.R408W/not identified | 2.94 | -1.21 | 9.24 | 737 | 0.14 | 0.12 / 0.05 | No | 37 | p.D415N/not identified | 4.15 | 1.27 | 11.05 | 819 | -0.59 | 0.17 / 0.06 | No |
| 34 | p.R408W/p.V245A | 4.65 | 3.35 | 10.13 | 761 | 0.41 | 0.15 / 0.05 | No | 38 | p.A403V/not identified | 3.6 | 0.05 | 9.04 | 759 | -1.12 | 0.13 / 0.06 | No |
| 35 | p.R297H/not identified | 3.77 | 1 | 13.4 | 800 | 0.6 | 0.15 / 0.05 | No | 41 | p.A403V/IVS10-11g>a | 3.58 | 0 | 12.22 | 787 | 1.63 | 0.18 / 0.07 | No |
| 39 | p.R408W/p.V245A | 3.18 | -0.57 | 9.55 | 728 | 0.71 | 0.17 / 0.06 | No | 42 | A403V/A300S | 3.52 | -0.13 | 10.25 | 774 | -0.16 | 0.18 / 0.05 | No |
| 40 | p.R408W/p.I269L | 3 | -1.05 | 8.17 | 722 | -0.61 | 0.18 / 0.06 | No | 45* | p.P281L/p.A403V | 3.83 | 0.56 | 9.58 | 777 | -1 | 0.19 / 0.06 | No |
| 43 | p.R408W/p.V245A | 3.99 | 1.59 | 9.25 | 730 | 0.34 | 0.25 / 0.05 | No | 48* | p.R408W/p.A300S | 3.55 | -0.06 | 11.53 | 760 | 1.78 | 0.24 / 0.08 | No |
| 44^*^ | p.R408W/p.S87R | 2.97 | -1.13 | 8.9 | 724 | 0.12 | 0.22 / 0.05 | No | 49 | p.R408W/p.A300S | 3.55 | -0.06 | 10.22 | 779 | -0.34 | 0.24 / 0.09 | No |
| 46 | p.P281L/p.A403V | 3.2 | -0.52 | 10.18 | 759 | 0.51 | 0.24 / 0.05 | No | 50 | p.R408W/p.A300S | 3.2 | -0.85 | 8.62 | 724 | -0.61 | 0.25 / 0.06 | No |
| 47^*^ | p.R408W/p.D415N | 3 | -1.05 | 13.58 | 820 | 1.93 | 0.24 / 0.07 | No | 52 | p.R408W/p.R297H | 3.34 | -0.53 | 11.65 | 802 | 0.52 | 0.32 / 0.05 | No |
| 51 | p.R158Q/p.Y414C | 3.4 | 0.02 | 12.1 | 764 | 2.23 | 0.26 / 0.08 | No |  |  |  |  |  |  |  |  |  |
| Wild-type individuals (no rs113883650 variant of the LAT1 gene) | | | | | | | | | | | | | | | | | |
| Girls | | | | | | | | | Boys | | | | | | | | |
| 55 | p.R408W/not identified | 3.77 | 1 | 9.64 | 763 | -0.11 | 0.18 / 0.08 | Yes | 53 | p.R408W/p.R158Q | 3.25 | -0.73 | 10.07 | 748 | 0.45 | 0.12 / 0.12 | Yes |
| 62 | p.R408W/p.R408W | 3.49 | 0.26 | 10.79 | 772 | 0.76 | 0.21 / 0.18 | Yes | 54 | p.R408W/p.R408W | 4.3 | 1.61 | 9.23 | 756 | -0.81 | 0.16 / 0.1 | Yes |
| 65 | p.R408W/p.P281L | 4.12 | 1.94 | 8.51 | 756 | -1.05 | 0.21 / 0.15 | Yes | 56 | p.R408W/p.R408W | 3.17 | -0.91 | 8.82 | 777 | -1.86 | 0.18 / 0.12 | Yes |
| 67 | p.R408W/p.R408W | 3.13 | -0.7 | 9.03 | 726 | 0.21 | 0.22 / 0.19 | Yes | 57 | p.R408W/p.Y414C | 3 | -1.29 | 9.38 | 782 | -1.36 | 0.018 / 0.11 | Yes |
| 69 | p.R408W/p.R408W | 3.2 | -0.52 | 8.08 | 740 | -1.13 | 0.23 / 0.18 | Yes | 58 | p.R408W/p.R408W | 3.7 | 0.27 | 9.19 | 738 | -0.32 | 0.18 / 0.1 | Yes |
| 71 | p.R408W/p.R408W | 3.15 | -0.65 | 8.57 | 733 | -0.45 | 0.23 / 0.13 | Yes | 59 | p.R408W/p.R408W | 3.66 | 0.18 | 9.74 | 750 | -0.01 | 0.19 / 0.22 | Yes |
| 73 | p.R408W/p.R408W | 3.43 | 0.1 | 10.27 | 753 | 0.76 | 0.24 / 0.11 | Yes | 60 | p.R408W/p.R408W | 2.73 | -1.9 | 11.6 | 776 | 1.31 | 0.2 / 0.2 | Yes |
| 79 | p.R408W/p.R408W | 2.78 | -1.64 | 12 | 765 | 2.09 | 0.25 / 0.14 | Yes | 61 | p.R408W/p.R408W | 3.25 | -0.73 | 9.14 | 704 | 0.75 | 0.21 / 0.21 | Yes |
|  |  |  |  |  |  |  |  |  | 63 | p.R408W/IVS10-11g>a | 2.75 | -1.85 | 10.85 | 782 | 0.27 | 0.21 / 0.12 | Yes |
|  |  |  |  |  |  |  |  |  | 64 | p.R408W/p.R158Q | 3 | -1.29 | 8.64 | 752 | -1.40 | 0.21 / 0.15 | Yes |
|  |  |  |  |  |  |  |  |  | 66 | p.R408W/IVS10-11g>a | 3.1 | -1.07 | 9.6 | 745 | -0.03 | 0.22 / 0.11 | Yes |
|  |  |  |  |  |  |  |  |  | 68 | p.R408W/p.F39del | 3.39 | -0.42 | 9.51 | 754 | -0.42 | 0.22 / 0.15 | Yes |
|  |  |  |  |  |  |  |  |  | 70 | p.R408W/p.R408W | 4.6 | 2.28 | 10.02 | 744 | 0.52 | 0.23 / 0.19 | Yes |
|  |  |  |  |  |  |  |  |  | 72 | p.R408W/not identified | 3.95 | 0.83 | 9.91 | 768 | -0.37 | 0.23 / 0.15 | Yes |
|  |  |  |  |  |  |  |  |  | 74 | p.R408W/p.R408W | 3.32 | -0.58 | 9.31 | 764 | -0.95 | 0.25 / 0.19 | Yes |
|  |  |  |  |  |  |  |  |  | 75 | p.R408W/p.R408W | 3 | -1.29 | 10.02 | 752 | 0.26 | 0.27 / 0.15 | Yes |
|  |  |  |  |  |  |  |  |  | 76 | p.R408W/p.R408W | 3.64 | 1.14 | 10.1 | 798 | -1.01 | 0.27 / 0.17 | Yes |
|  |  |  |  |  |  |  |  |  | 77 | p.R408W/p.R408W | 3.4 | -0.4 | 9.45 | 7.32 | 0.2 | 0.24 / 0.15 | Yes |
|  |  |  |  |  |  |  |  |  | 78 | p.R408W/IVS12+1g>a | 3.48 | -0.22 | 9.88 | 767 | -0.37 | 0.24 / 0.18 | Yes |
| 80 | p.R408W/p.A403V | 2.7 | -1.85 | 9.9 | 715 | 1.46 | 0.36 / 0.05 | No | 82 | p.R408W/p.A403V | 3 | -1.29 | 9.67 | 735 | 0.38 | 0.17 / 0.02 | No |
| 81 | p.R297H/not identified | 3.45 | 0.15 | 9.04 | 728 | 0.17 | 0.28 / 0.12 | No | 84 | p.R408W/not identified | 3.5 | -0.18 | 9.55 | 795 | -1.52 | 0.11 / 0.04 | No |
| 83 | p.A300S/p.I306V | 2.89 | -1.34 | 9.29 | 710 | 0.94 | 0.2 / 0.01 | No | 85 | p.I306V/IVS10-11g>a | 3.65 | 0.16 | 11 | 785 | 0.35 | 0.12 / 0.07 | No |
| 87 | IVS10-11g>a/p.I306V | 2.69 | -1.88 | 11.08 | 748 | 1.71 | 0.19 / 0.07 | No | 86 | p.A403V/p.I306V | 3.35 | -0.51 | 11.3 | 786 | 0.65 | 0.16 / 0.05 | No |
| 90 | p.V245A/p.R408W | 3.57 | 0.47 | 9.72 | 750 | 0.30 | 0.18 / 0.05 | No | 88 | p.R408W/p.R53H | 4.5 | 2.06 | 10.8 | 791 | -0.05 | 0.17 / 0.05 | No |
| 94 | p.R408W/p.R297H | 2.82 | -1.53 | 8.54 | 740 | -0.65 | 0.23 / 0.06 | No | 89 | p.I269L/p.R158Q | 3.9 | 0.72 | 12.27 | 798 | 1.31 | 0.18 / 0.05 | No |
| 99 | p.R408W/p.L358F | 3.56 | 0.44 | 10.16 | 778 | 0.01 | 0.22 / 0.07 | No | 91 | p.R408W/p.A300S | 3.05 | -1.18 | 10.32 | 742 | 0.96 | 0.18 / 0.04 | No |
| 102 | p.R408W/p.A300S | 3.31 | -0.22 | 9.69 | 754 | 0.16 | 0.24 / 0.06 | No | 92 | p.D394H/p.R408W | 3.66 | 0.18 | 10.16 | 787 | -0.64 | 0.19 / 0.09 | No |
| 103 | p.L358F/p.S16>XfsX1 | 3.43 | 0.1 | 9.43 | 767 | -0.41 | 0.23 / 0.06 | No | 93 | p.A300S/p.R408W | 3.18 | -0.89 | 10.48 | 740 | 1.22 | 0.21 / 0.07 | No |
| 104 | p.R408W/p.D415N | 3.6 | 0.55 | 10.17 | 778 | 0.02 | 0.26 / 0.07 | No | 95 | p.R297H/p.R408W | 3.2 | -0.85 | 9.1 | 725 | -0.02 | 0.22 / 0.05 | No |
| 105 | p.R408W/p.A403V | 2.97 | -1.13 | 9.26 | 750 | -0.17 | 0.31 / 0.06 | No | 96 | p.R408W/p.A403V | 3.67 | 0.2 | 10.15 | 777 | -0.36 | 0.24 / 0.06 | No |
| 107 | p.R408W/p.I306V | 3.23 | -0.44 | 9 | 766 | -0.80 | 0.3 / 0.05 | No | 97 | p.R408W/p.A300S | 3.08 | -1.11 | 8.87 | 724 | -0.28 | 0.21 / 0.05 | No |
| 108 | p.R408W/p.R297H | 3.15 | -0.65 | 8.88 | 762 | -0.82 | 0.29 / 0.05 | No | 98 | p.R297H/p.R297H | 3.83 | 0.56 | 10.97 | 782 | 0.41 | 0.22 / 0.05 | No |
| 109 | p.R408W/p.I306V | 3.28 | -0.3 | 10.3 | 748 | 0.93 | 0.29 / 0.07 | No | 100 | p.V230I/p.R408W | 3.58 | 0 | 10.05 | 782 | -0.62 | 0.23 / 0.06 | No |
|  |  |  |  |  |  |  |  |  | 101 | p.A300S/IVS10-11g>a | 3.1 | -1.07 | 9.75 | 763 | -0.40 | 0.22 / 0.06 | No |
|  |  |  |  |  |  |  |  |  | 106 | p.P211T/p.R261Q | 3 | -1.29 | 9.38 | 730 | 0.18 | 0.25 / 0.11 | No |
